# Embolization of aortic valve leaflet during valve-in-valve transcatheter aortic valve implantation: a case report

**DOI:** 10.1093/ehjcr/ytaa010

**Published:** 2020-02-12

**Authors:** Sudhakar Kinthala, Poovendran Saththasivam, Abistanand Ankam, Sudhakar Sattur

**Affiliations:** y1 Department of Anesthesiology, Guthrie Robert Packer Hospital, Sayre, PA 18840, USA; y2 Department of Cardiology, Guthrie Robert Packer Hospital, Sayre, PA 18840, USA

**Keywords:** Aortic stenosis, Transcatheter aortic valve implantation, Valve leaflet avulsion, Embolization, Valve-in-valve, Vascular complications, Case report

## Abstract

**Background:**

Aortic stenosis (AS) is one of the most common valvular disorders worldwide. An increasing number of transcatheter aortic valve implantation (TAVI) procedures are being performed yearly for managing AS. This, along with the occurrence of common complications, makes timely diagnosis essential to manage rare complications and improve patient outcomes.

**Case summary:**

We present a case of a 77-year-old Caucasian male with severe AS with a dysfunctional bioprosthetic valve following previous surgical valve replacement. During valve-in-valve TAVI, we noted bioprosthetic valve leaflet avulsion and embolization causing a major vascular occlusion that resulted in vascular insufficiency of the left lower extremity. This condition was managed successfully via immediate diagnosis using transoesophageal echocardiogram, angiogram, and vascular surgical intervention for retrieving the embolized valve to re-establish circulation.

**Discussion:**

To our knowledge, this is the first case of aortic valve leaflet embolization during TAVI resulting in significant vascular insufficiency. Vascular complications are common during TAVI. However, not all vascular complications are the same. Our case highlights an embolic vascular complication from an avulsed prosthetic material during a challenging valve-in-valve TAVI procedure.

**Learning points**

- An increasing number of transcatheter aortic valve implantation (TAVI) procedures are being performed, making it important to recognize and manage rare complications to improve patient outcomes.
- Vascular complications are common during TAVI; however, not all vascular complications are the same.
- During TAVI, when a mobile echogenic mass was found in the left ventricle and ascending aorta by echocardiogram, associated with acute aortic insufficiency, possible valve avulsion with potential embolization should be suspected. A workup should be performed to locate the embolus with interventions for embolectomy and revascularization.

## Introduction

Critical aortic stenosis affects an estimated 4.6% of individuals over 77 years of age.^1^ It is considered reasonable to perform valve-in-valve transcatheter aortic valve implantation (TAVI) for severely symptomatic patients with bioprosthetic valve stenosis or regurgitation who have high risk of re-operation and in whom improvement in haemodynamics is anticipated.^2^ Common complications during the TAVI procedure include major vascular complications, bleeding, stroke, coronary obstruction, and arrhythmias.^3^^,^^4^ Thus far, few cases of native or prosthetic valve leaflet avulsion without embolization during TAVI have been reported; these patients were managed conservatively.^5–8^ We report a case of bioprosthetic valve leaflet avulsion and embolization causing major vascular occlusion during the valve-in-valve TAVI procedure. To our knowledge, this is the first case of valve leaflet avulsion and embolization that was managed successfully by immediate diagnosis and vascular surgical intervention.

## Timeline

**Table:** 

Time	Events
0 min	Valve-in-valve transcatheter aortic valve implantation started under general anaesthesia under transoesophageal echocardiogram (TOE) guidance.
60 min	Nosecone advanced across the bioprosthetic valve. Patient became hypotensive. TOE showed a large mobile echogenic mass that appeared to be moving back and forth from the left ventricle to the aorta through the annulus.
70 min	No pericardial effusion was noted on TOE. Severe intra-valvular regurgitation was noted. No response was observed to intravenous vasopressors, inotropes, and rapid ventricular pacing to improve haemodynamic stability. The large mobile mass was no longer seen. Evolut R valve deployment.
90 min	Absence of distal left lower extremity pulses was noted. Angiography showed a large filling defect at the level of the left common femoral artery bifurcation.
180 min	Open exploration of the femoral artery and embolectomy was performed. Leaflet of previous bioprosthetic valve was removed. Left lower extremity circulation was re-established.

## Case presentation

**Figure 1:**
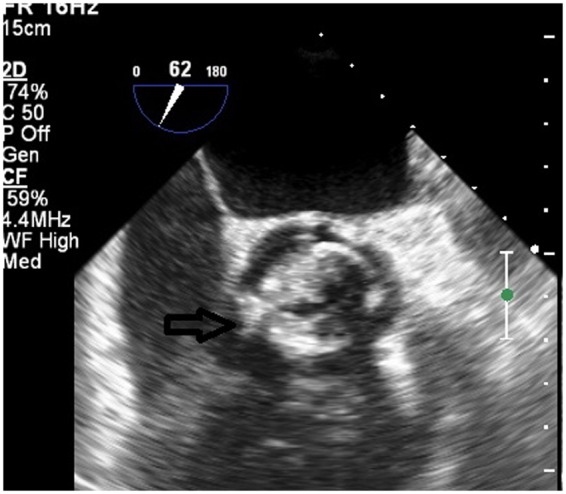
Transoesophageal echocardiogram mid-oesophageal short-axis view showing bioprosthetic aortic valve with severe stenosis and calcification (arrow pointing).

**Figure 2:**
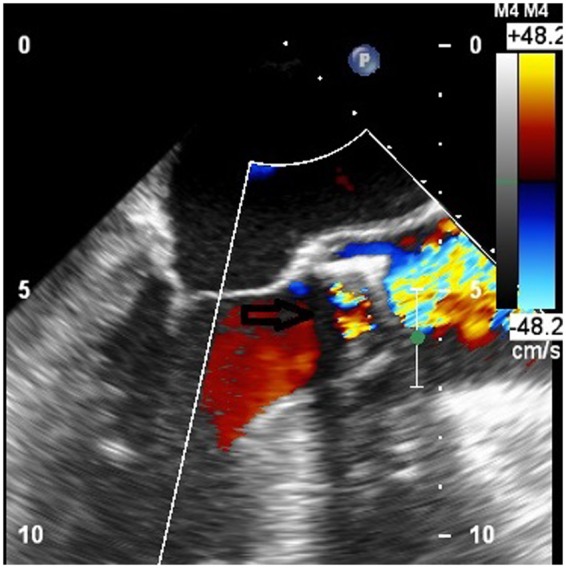
Transoesophageal echocardiogram mid-oesophageal long-axis view showing turbulent flow through the bioprosthetic aortic valve (arrow pointing).

We present the case of a 77-year-old Caucasian male with a medical history of hypertension, hyperlipidaemia, hypothyroidism, triple vessel coronary artery bypass, and surgical aortic valve replacement with a 27-mm Mosaic Ultra valve 10 years prior. He developed progressively worsening shortness of breath (New York Heart Association Class III symptoms) on mild exertion for 1 month. He denied dizziness or lightheadedness, chest pain, or palpitations. On cardiac auscultation, a Grade IV mid-systolic murmur was audible along the upper right sternal border and was further diagnosed with severe bioprosthetic valve stenosis. Transoesophageal echocardiogram (TOE) showed left ventricular ejection fraction of 35–40%, severely calcified and restricted mobility of the bioprosthetic valve leaflets (*Figure 1* and Supplementary material online, *Video S1*). Doppler spectral profile showed low-flow, low-gradient aortic bioprosthetic stenosis with mean gradient of 34 mmHg (*Figure 2* and Supplementary material online, *Video S2*) and valve area of 0.6 cm^2^. No findings consistent with endocarditis were noted. Results of blood investigations revealed, haemoglobin 11 gm/dL and otherwise normal.

As a part of the workup 4 weeks prior to scheduled TAVI, the patient underwent a coronary angiogram and percutaneous intervention of the left-main/ostial circumflex with a drug-eluting stent. He was scheduled for a valve-in-valve TAVI because of his comorbid conditions including the need for re-operation and high Society of Thoracic Surgeons risk score. The use of a 26-mm Evolut R valve was recommended for the valve-in-valve TAVI. The procedure was performed under general anaesthesia under TOE guidance. An angiogram of the chest, abdomen, and pelvis confirmed the suitability of the iliofemoral vasculature for the TAVI procedure. Right femoral artery access was obtained under fluoroscopic guidance. Two Perclose devices were used to preclose the arterial access. The arterial access was upgraded to a 14-Fr cook sheath. A 6-Fr arterial sheath was placed through the left femoral artery after confirming suitability under fluoroscopy. A 6-Fr pigtail catheter was placed in the ascending aorta. The patient was heparinized to maintain activated clotting time >300 s during the procedure.

**Figure 3:**
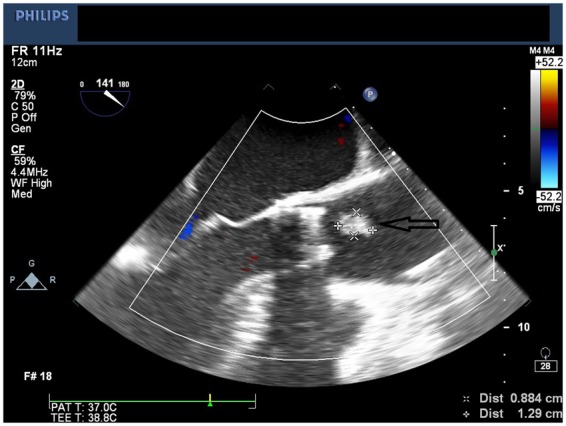
Arrow pointing large mobile echogenic mass (9 mm × 13 mm) seen on mid-oesophageal long-axis view.

**Figure 4:**
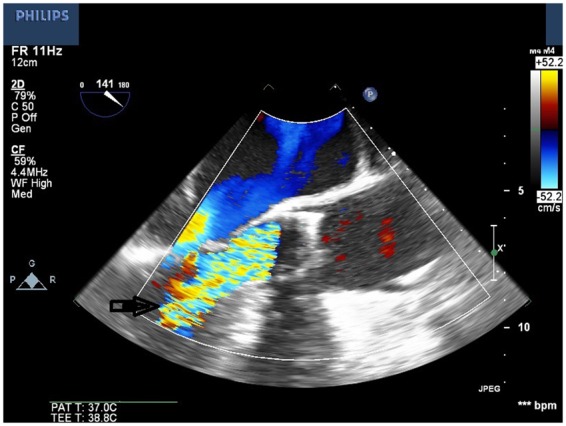
Mid-oesophageal long-axis view showing severe intravalvular regurgitation.

**Figure 5:**
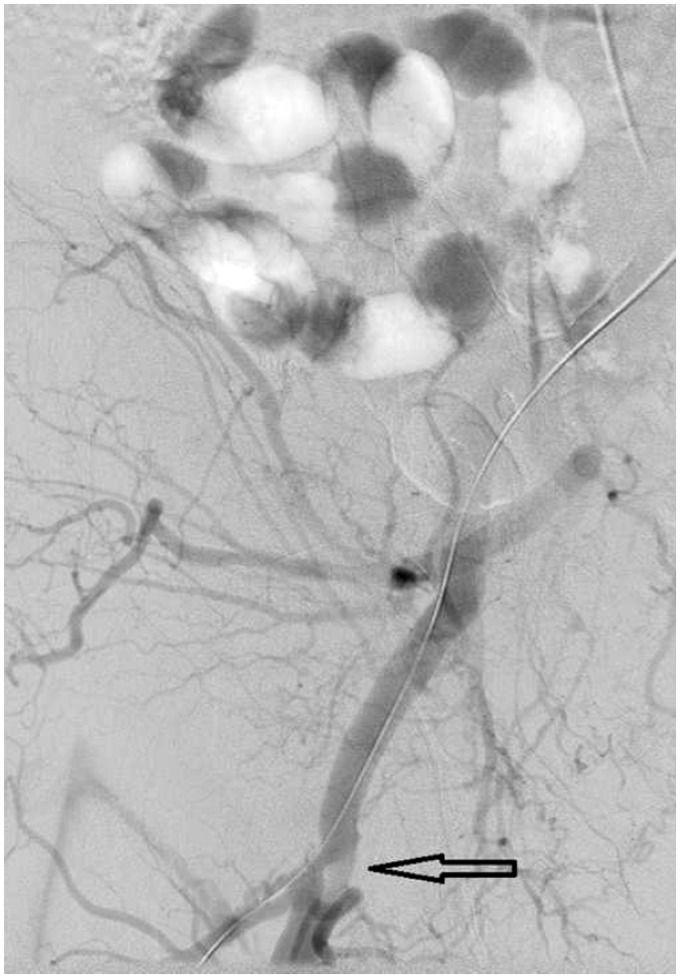
Angiography showing a large filling defect at the femoral bifurcation level (arrow pointing).

**Figure 6:**
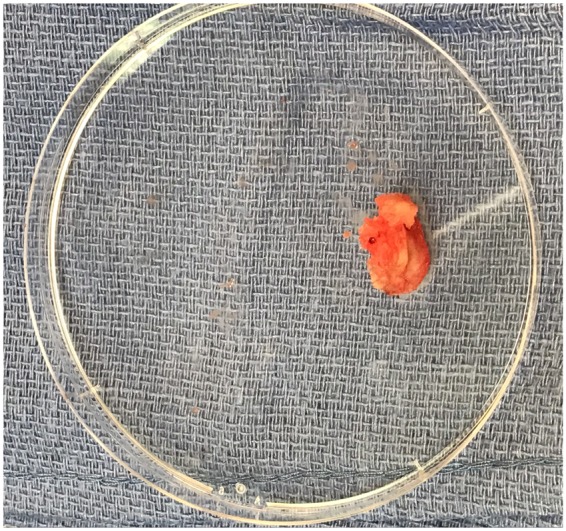
A leaflet/fragment of the previous bioprosthetic valve retrieved through embolectomy, measuring 1.0 cm × 1.4 cm.

The bioprosthetic valve was crossed using a 4-Fr Amplatz Left 1 (AL1) catheter and a 0.035-inch straight wire. The AL1 catheter was exchanged to a 5-Fr pigtail catheter. A 0.035-inch pre-shaped Amplatz wire was introduced and positioned in the left ventricular apex. Over this wire, a 26-mm Medtronic valve was introduced. Significant resistance was encountered when advancing the nosecone across the bioprosthetic valve. After the nosecone was introduced into the left ventricle (LV) apex, the patient became hypotensive. Therefore, TOE was performed to assess for pericardial effusion. Although no pericardial effusion was noted, a large mobile echogenic mass (9 mm × 13 mm) was seen that appeared to be moving back and forth from the LV to the aorta through the annulus (*Figure 3* and Supplementary material online, *Video S3*). Further, severe intravalvular regurgitation was noted (*Figure 4* and Supplementary material online, *Video S4*). The patient was administered intravenous vasopressors, inotropes, and rapid ventricular pacing to improve haemodynamic stability. The large mobile mass was no longer seen across the aortic annulus. We proceeded further by deploying the Evolut R valve in a pre-selected coplanar view. Post-valve deployment, haemodynamic stability was attained. Both TOE and ascending aortogram confirmed no paravalvular leak. The delivery sheath was removed from the body and exchanged to a 14-Fr sheath. The Perclose sutures were used to close the entry site. A subsequent right iliofemoral angiogram showed no evidence of dissection at the entry site with preserved flow beyond it. The left common femoral arterial access was closed using a Perclose suture. Distal left lower extremity pulses were absent at the end of the procedure; therefore, a limited left iliofemoral angiogram was performed from the contralateral access. Angiography showed a large filling defect at the femoral bifurcation level (*Figure 5* and Supplementary material online, *Video S5*). We suspected that the filling defect was the embolized prosthetic valve leaflet. Open exploration of the femoral artery and an embolectomy were performed. A leaflet/fragment of the previous bioprosthetic valve was removed (*Figure 6*).

The post-operative course was uneventful; no neurological deficits were noted, and the patient was discharged home on post-operative Day 2. At 1-month follow-up, the patient remained without symptoms of heart failure and able to take care of daily activities of living comfortably.

## Discussion

Vascular complications are not uncommon during a TAVI procedure.^4^^,^^9^^,^^10^ However, the aetiology of most of these complications is thrombotic and is related to focal vessel injury at the vessel entry site. Femoral artery embolic complications are rare during a TAVI procedure.

In our case, we suspect that during the act of crossing the prosthetic stenotic valve with the nosecone, the leaflet avulsed owing to mechanical trauma/shear stress that led to severe regurgitation and severe hypotension. Over the years, the role of TOE during TAVI procedures has decreased significantly owing to factors such as the need for general anaesthesia and increased length of stay.^11^ However, TOE could provide invaluable information that could help determine management during a TAVI procedure. In our patient, the use of TOE helped with the prompt detection of possible causes of haemodynamic instability and an incidental finding of a large mobile echogenic mass. These findings helped us to determine the strategy for treating the acute limb ischaemia noted at the end of the procedure. Thus far, few reported cases of aortic valve leaflet avulsion without embolization during transcatheter aortic valve replacement have been managed successfully via the percutaneous snare retrieval technique^5^ and endovascular snare technique.^8^ In our case, prior to proceeding with an open surgical procedure, a multidisciplinary team had a thorough discussion regarding the open vs. endovascular management options. Based on the TOE and angiogram findings, we pursued an open approach rather than an endovascular strategy to avoid fragmentation of the embolized prosthetic valve causing further distal embolization.

Our case also highlights the challenges faced in valve-in-valve TAVI procedures compared with native valve TAVI procedures. Bioprosthetic stenotic valves are difficult to cross compared with native valves. This could be related to several factors including the dense fibrous tissue matrix associated with stenotic prosthetic valves. To avoid avulsing the valve leaflet/tissue, valvuloplasty with an undersized balloon could be performed to decrease the resistance encountered with the crossing of prosthetic stenotic valve leaflets. The balloon-expandable Edwards S3 valve could have easier crossability through the stenotic valve, with shorter length of the nose cone and the valve which probably translate to less shearing force on the diseased valve leaflets, but it is unclear whether the use of a balloon-expandable Edwards TAVI valve would have led to a different outcome in our patient compared to a self-expanding Evolut R valve.

Vascular complications are common during a TAVI procedure. However, not all vascular complications are the same. Our case highlights an embolic vascular complication from an avulsed prosthetic material during a challenging valve-in-valve TAVI procedure. Our case also highlights the invaluable information obtained from a TOE that helped guide the strategy towards a favourable outcome for our patient.

## Lead author biography

**Figure:**
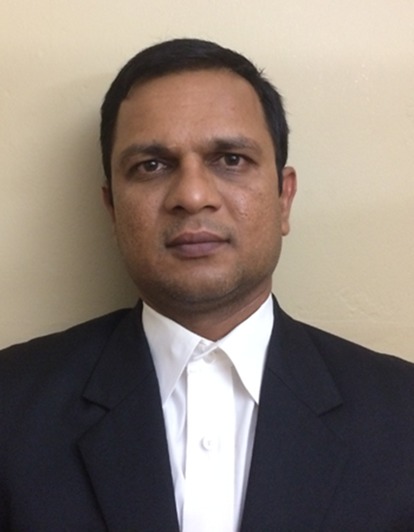


Sudhakar Kinthala is a diplomate of the American Board of Anesthesiology who works as an attending anaesthesiologist at Guthrie Robert Packer Hospital in Sayre, PA, USA. He has been providing clinical and academic anaesthesiology services for the past 16 years. He completed his residency in anaesthesiology from New York Presbyterian Brooklyn Methodist Hospital in collaboration with Weill Cornell Medicine. He is a fellowship trained Cardiac and neuroanaesthesiologist and board certified anaesthesiologist in India. He is actively involved in teaching residents and medical students. He has published widely in various journals. Dr Kinthala’s main areas of interest are cardiac and neuroanaesthesiology and perioperative patient safety.

## Supplementary material 

Supplementary material is available at *European Heart Journal - Case Reports* online.

**Slide sets:** A fully edited slide set detailing this case and suitable for local presentation is available online as Supplementary data.

**Consent:** The author/s confirm that written consent for submission and publication of this case report including image(s) and associated text has been obtained from the patient in line with COPE guidance. 

**Conflict of interest:** none declared. 

## Supplementary Material

ytaa010_Supplementary_Data
