# Supplementary material for: Embolization of aortic valve leaflet during valve-in-valve transcatheter aortic valve implantation: a case report
Source: Eur Heart J Case Rep. 2020 Feb 12;4(1):1–5. doi: 10.1093/ehjcr/ytaa010 (PMC7047047; doi:10.1093/ehjcr/ytaa010)
Supplement: ytaa010_Supplementary_Data [file ytaa010_supplementary_data.zip › ytaa010-suppl_data/R3-12-23-EHJ-CR-Slide-Set-TAVR.pptx]

## Slide 1
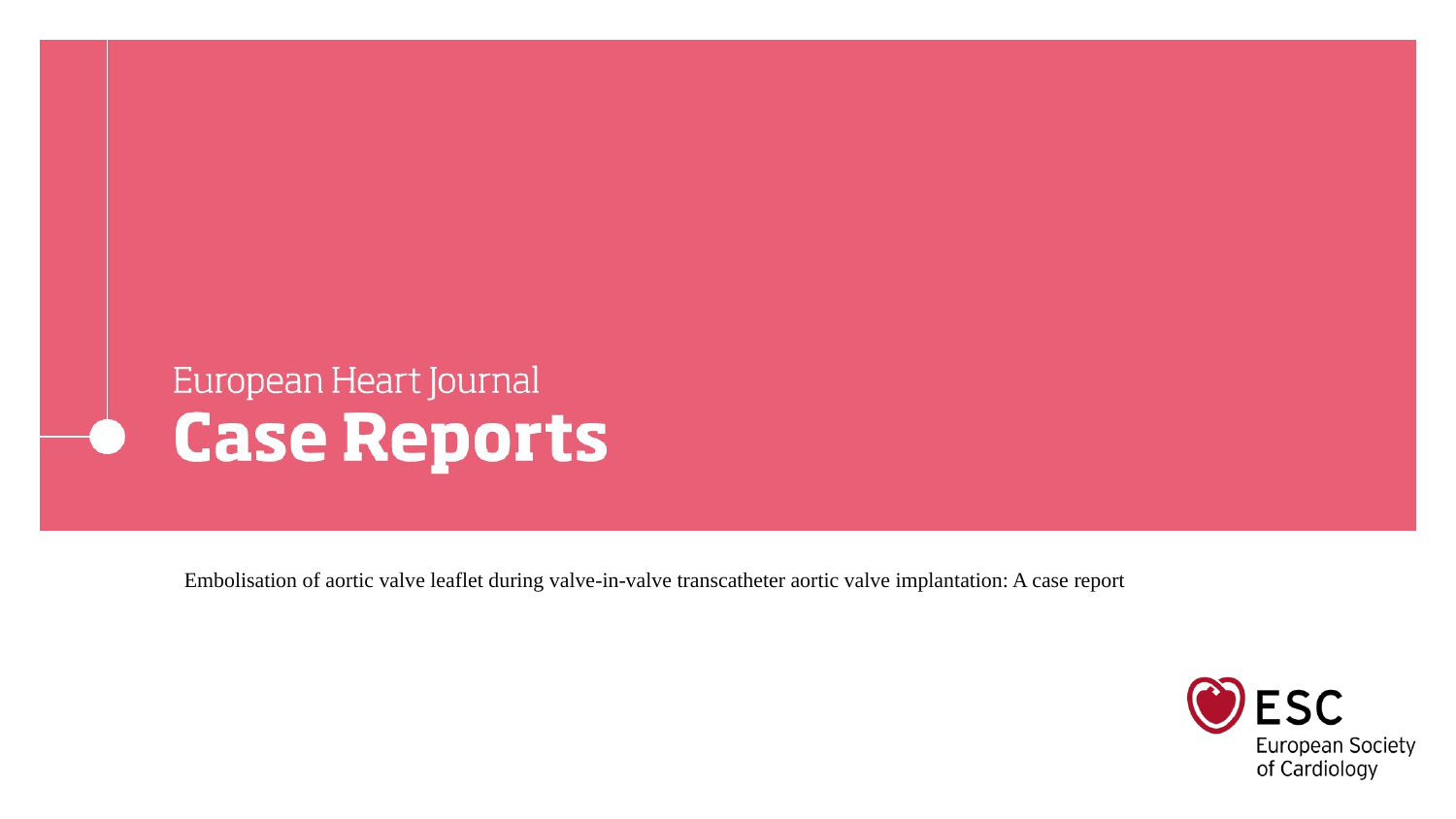

# Embolisation of aortic valve leaflet during valve-in-valve transcatheter aortic valve implantation: A case report

## Slide 2
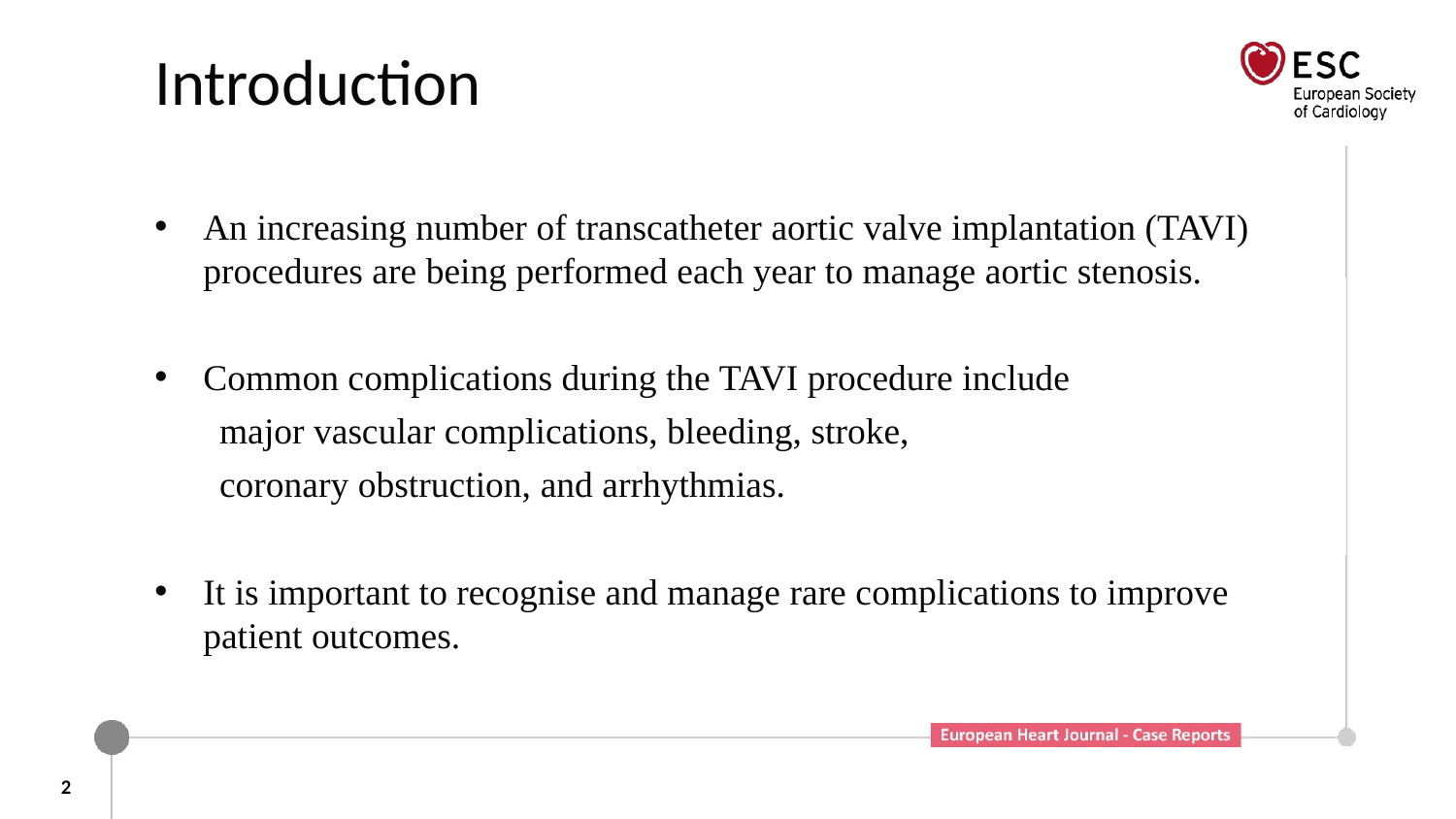

# Introduction
An increasing number of transcatheter aortic valve implantation (TAVI) procedures are being performed each year to manage aortic stenosis.
Common complications during the TAVI procedure include
 major vascular complications, bleeding, stroke,
 coronary obstruction, and arrhythmias.
It is important to recognise and manage rare complications to improve patient outcomes.
2

## Slide 3
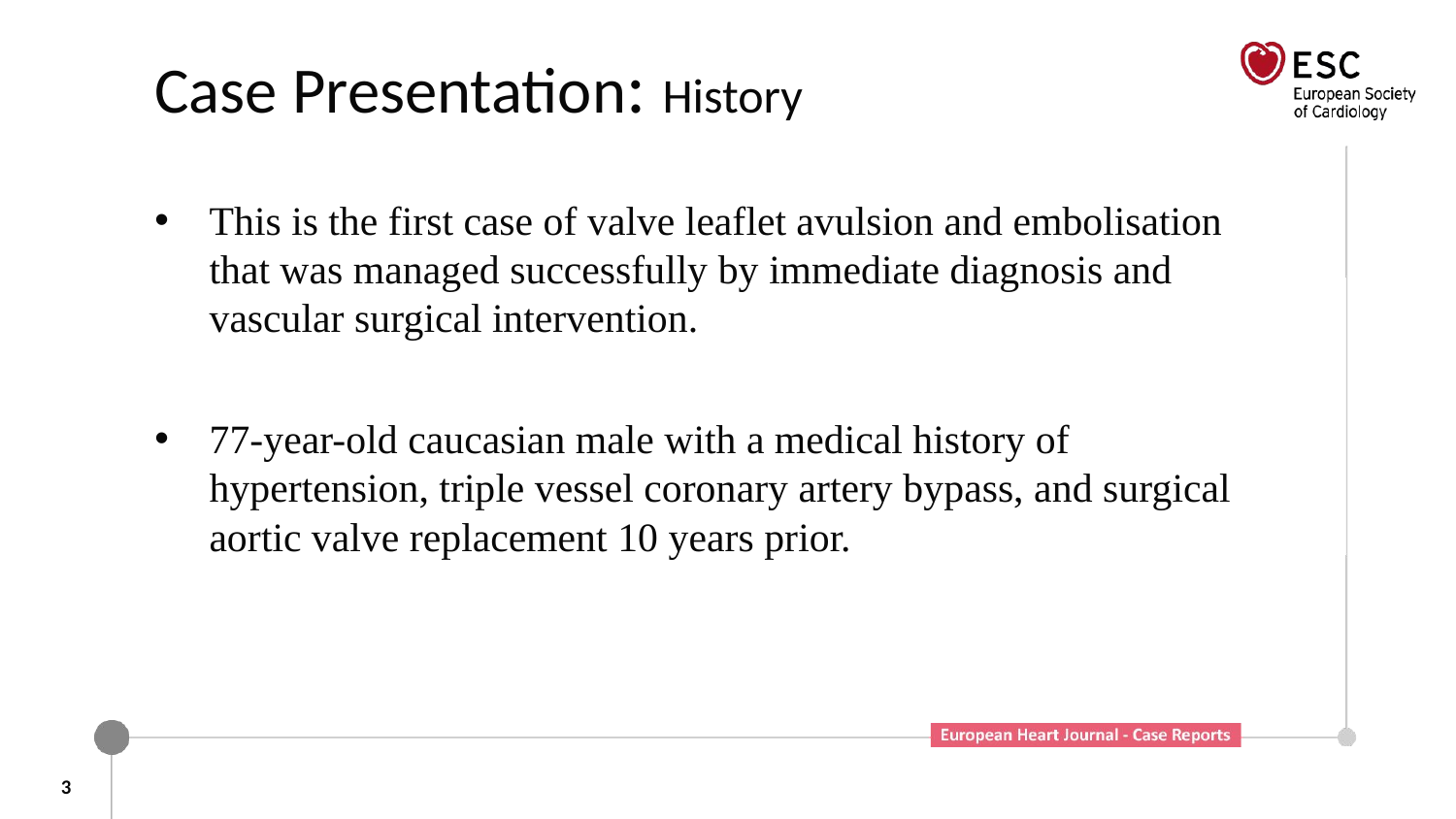

# Case Presentation: History
This is the first case of valve leaflet avulsion and embolisation that was managed successfully by immediate diagnosis and vascular surgical intervention.
77-year-old caucasian male with a medical history of hypertension, triple vessel coronary artery bypass, and surgical aortic valve replacement 10 years prior.
3

## Slide 4
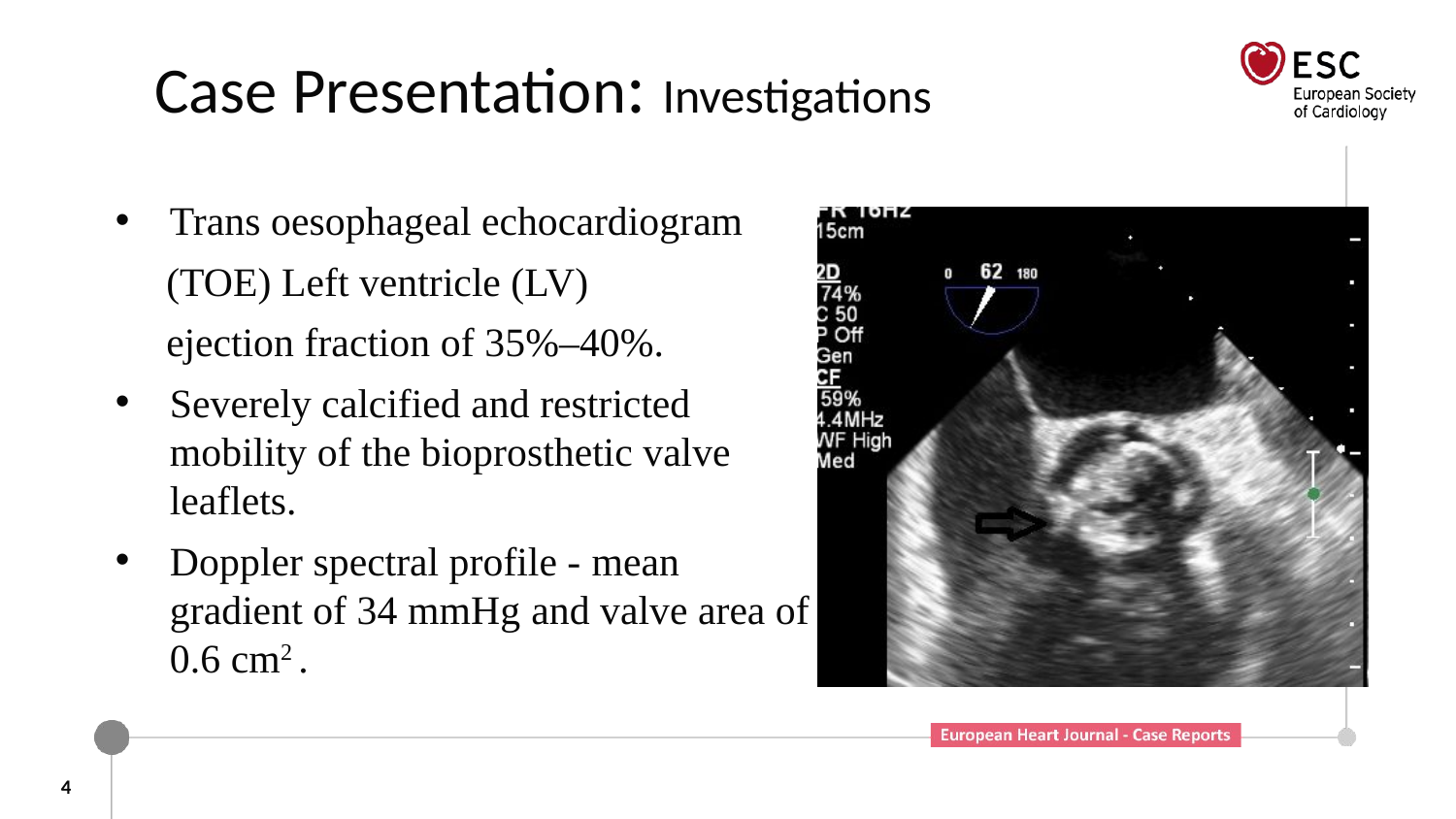

# Case Presentation: Investigations
Trans oesophageal echocardiogram
 (TOE) Left ventricle (LV)
 ejection fraction of 35%–40%.
Severely calcified and restricted mobility of the bioprosthetic valve leaflets.
Doppler spectral profile - mean gradient of 34 mmHg and valve area of 0.6 cm2 .
4

## Slide 5
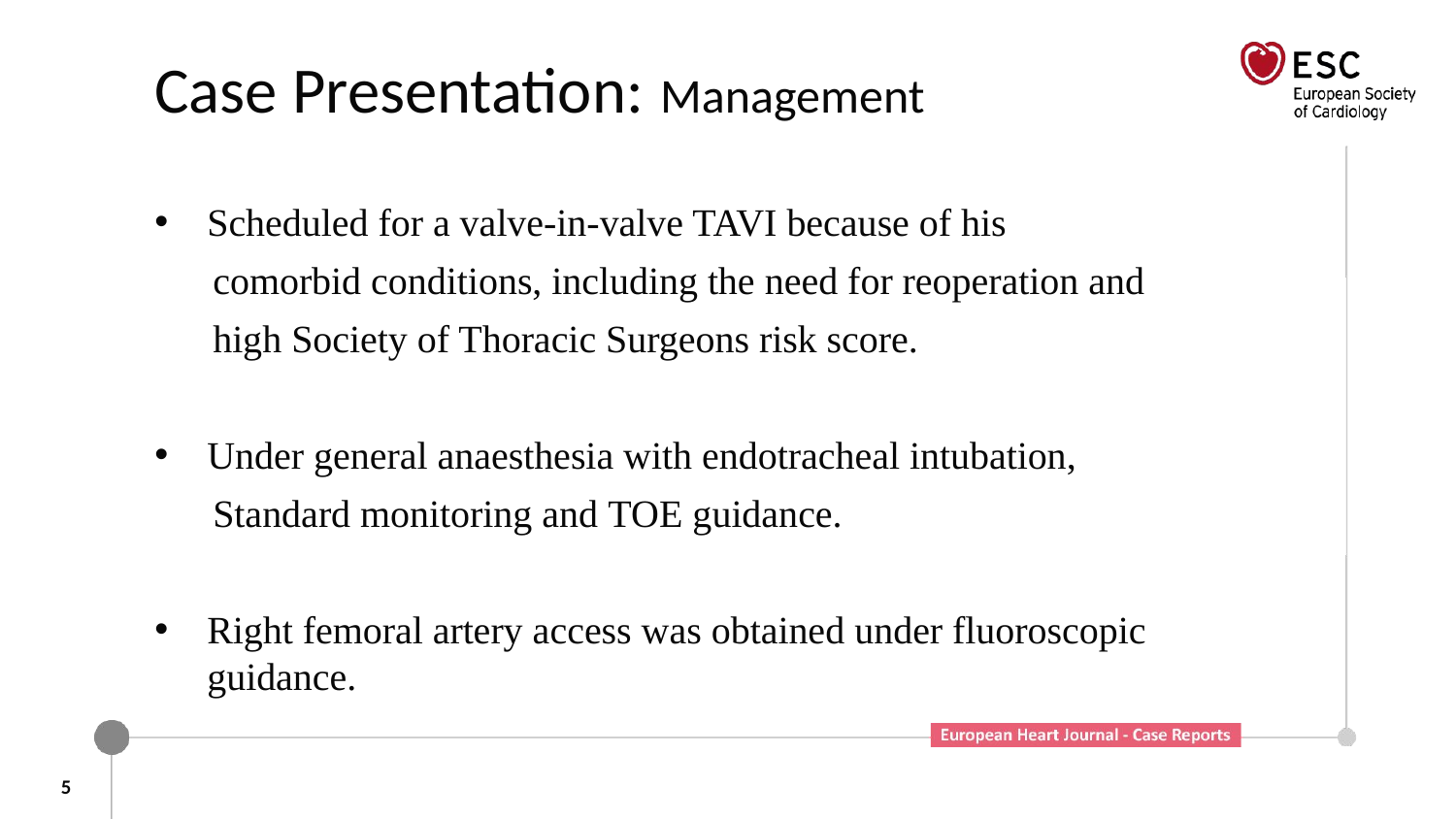

# Case Presentation: Management
Scheduled for a valve-in-valve TAVI because of his
 comorbid conditions, including the need for reoperation and
 high Society of Thoracic Surgeons risk score.
Under general anaesthesia with endotracheal intubation,
 Standard monitoring and TOE guidance.
Right femoral artery access was obtained under fluoroscopic guidance.
5

## Slide 6
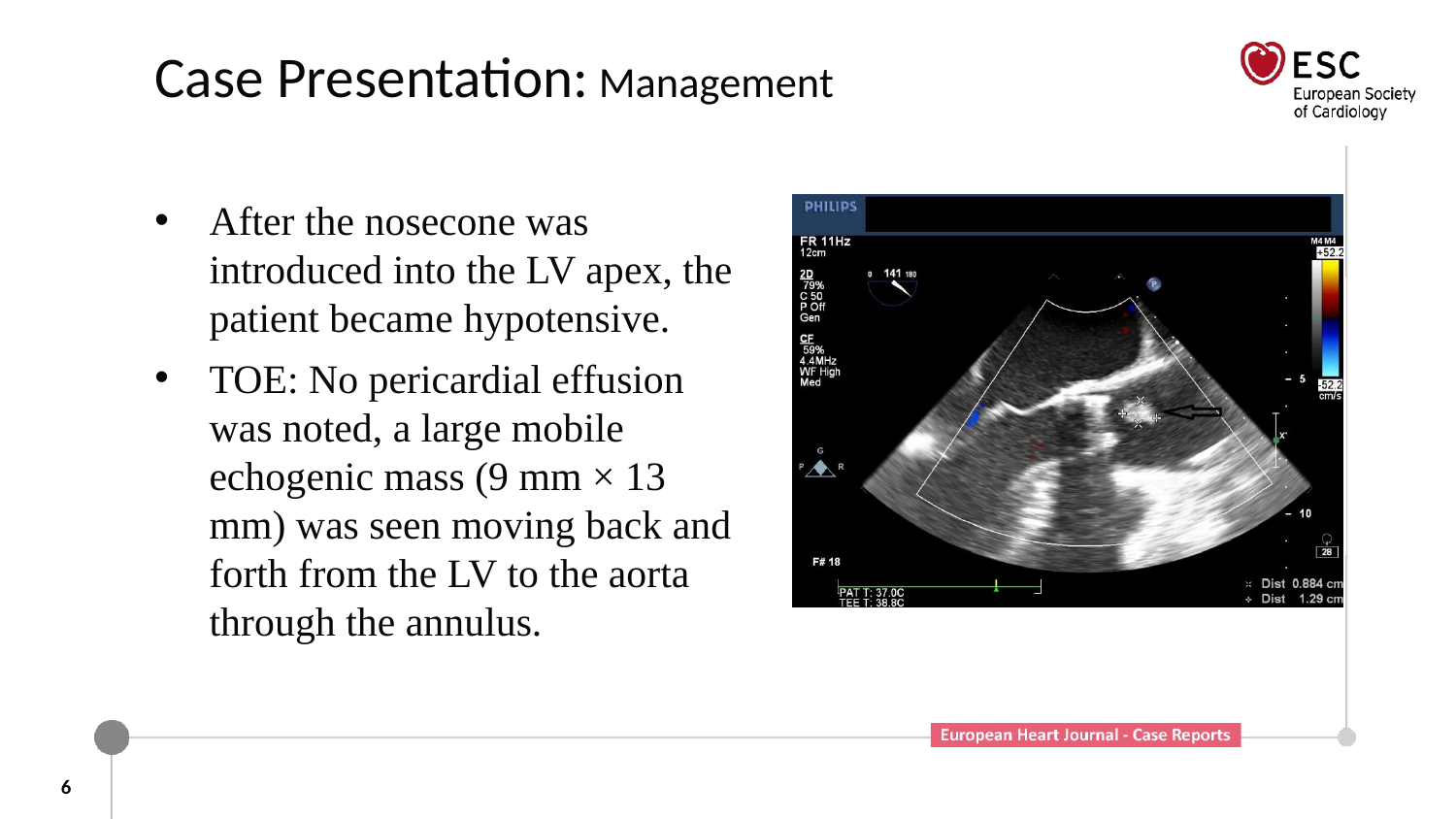

# Case Presentation: Management
After the nosecone was introduced into the LV apex, the patient became hypotensive.
TOE: No pericardial effusion was noted, a large mobile echogenic mass (9 mm × 13 mm) was seen moving back and forth from the LV to the aorta through the annulus.
6

## Slide 7
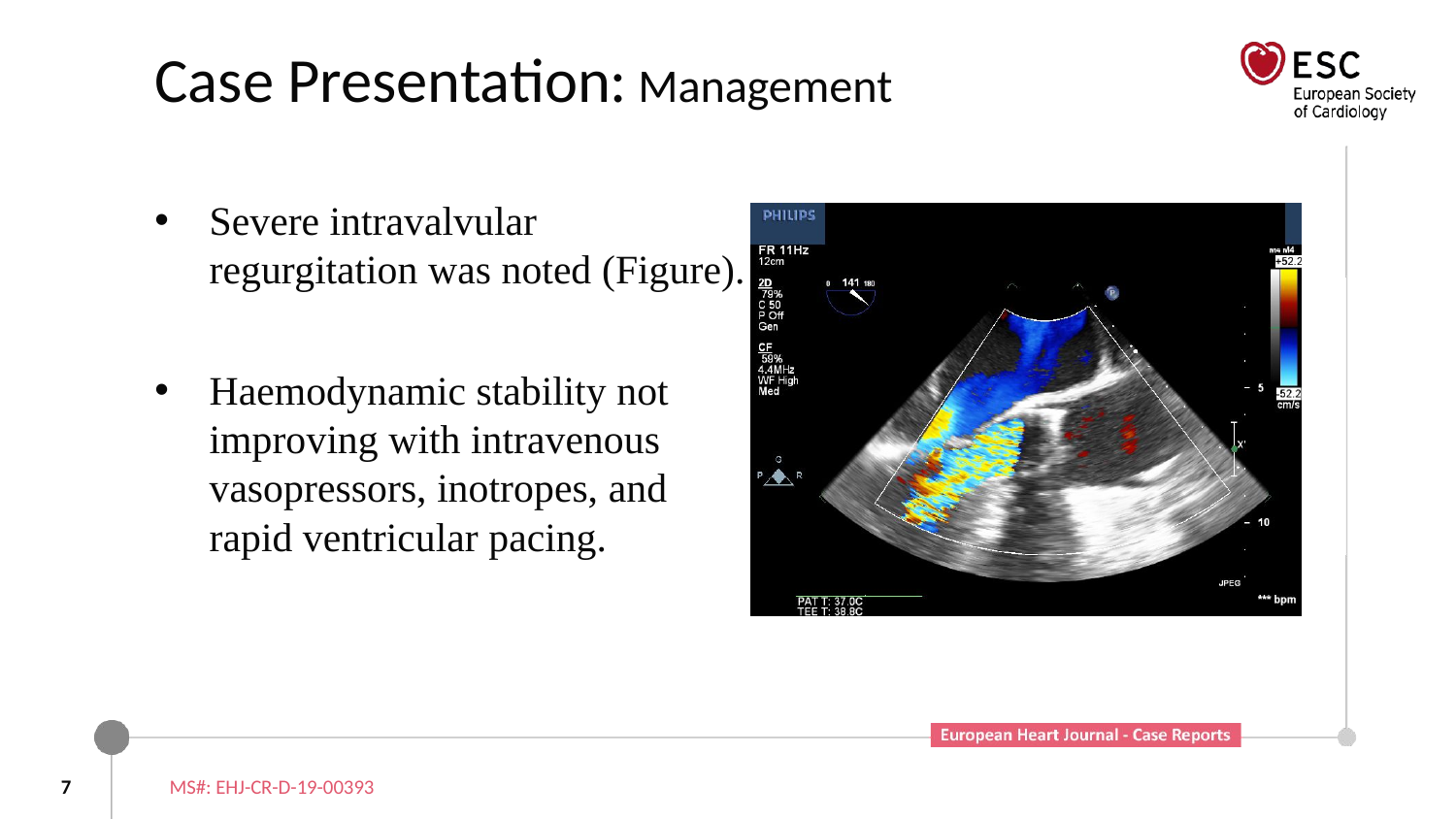

# Case Presentation: Management
Severe intravalvular regurgitation was noted (Figure).
Haemodynamic stability not improving with intravenous vasopressors, inotropes, and rapid ventricular pacing.
7
MS#: EHJ-CR-D-19-00393

## Slide 8
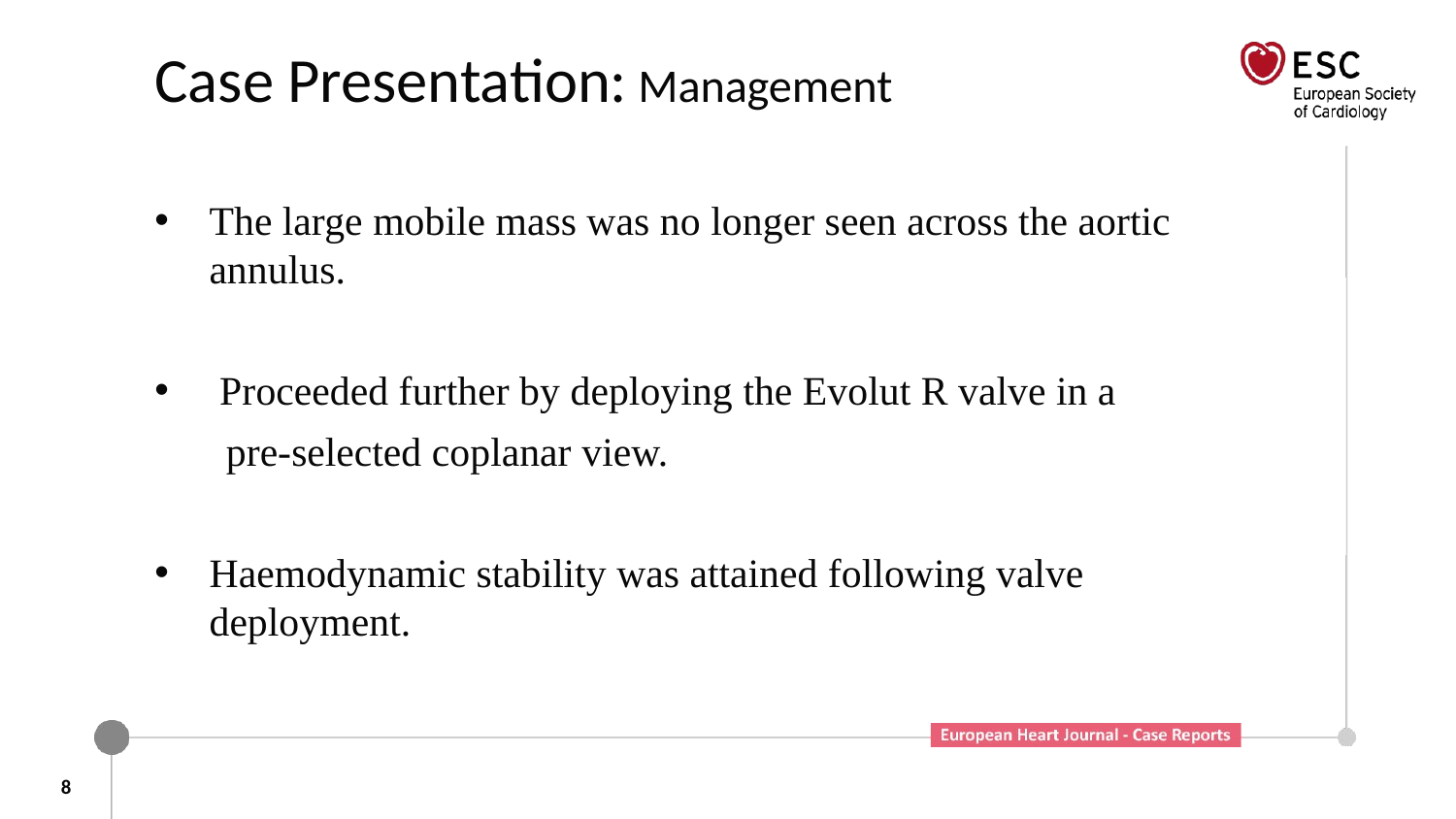

# Case Presentation: Management
The large mobile mass was no longer seen across the aortic annulus.
 Proceeded further by deploying the Evolut R valve in a
 pre-selected coplanar view.
Haemodynamic stability was attained following valve deployment.
8

## Slide 9
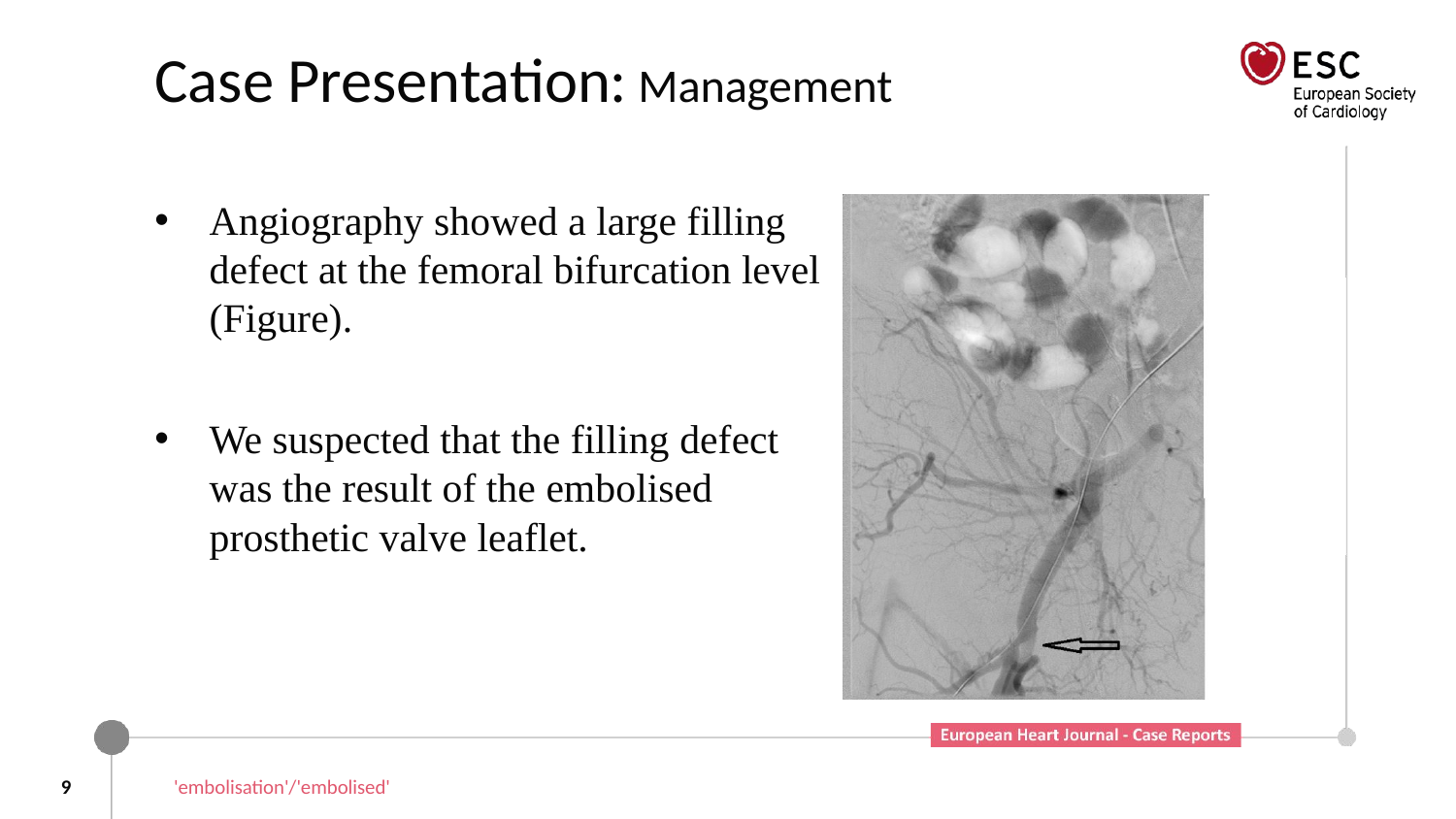

# Case Presentation: Management
Angiography showed a large filling defect at the femoral bifurcation level (Figure).
We suspected that the filling defect was the result of the embolised prosthetic valve leaflet.
9
 'embolisation'/'embolised'

## Slide 10
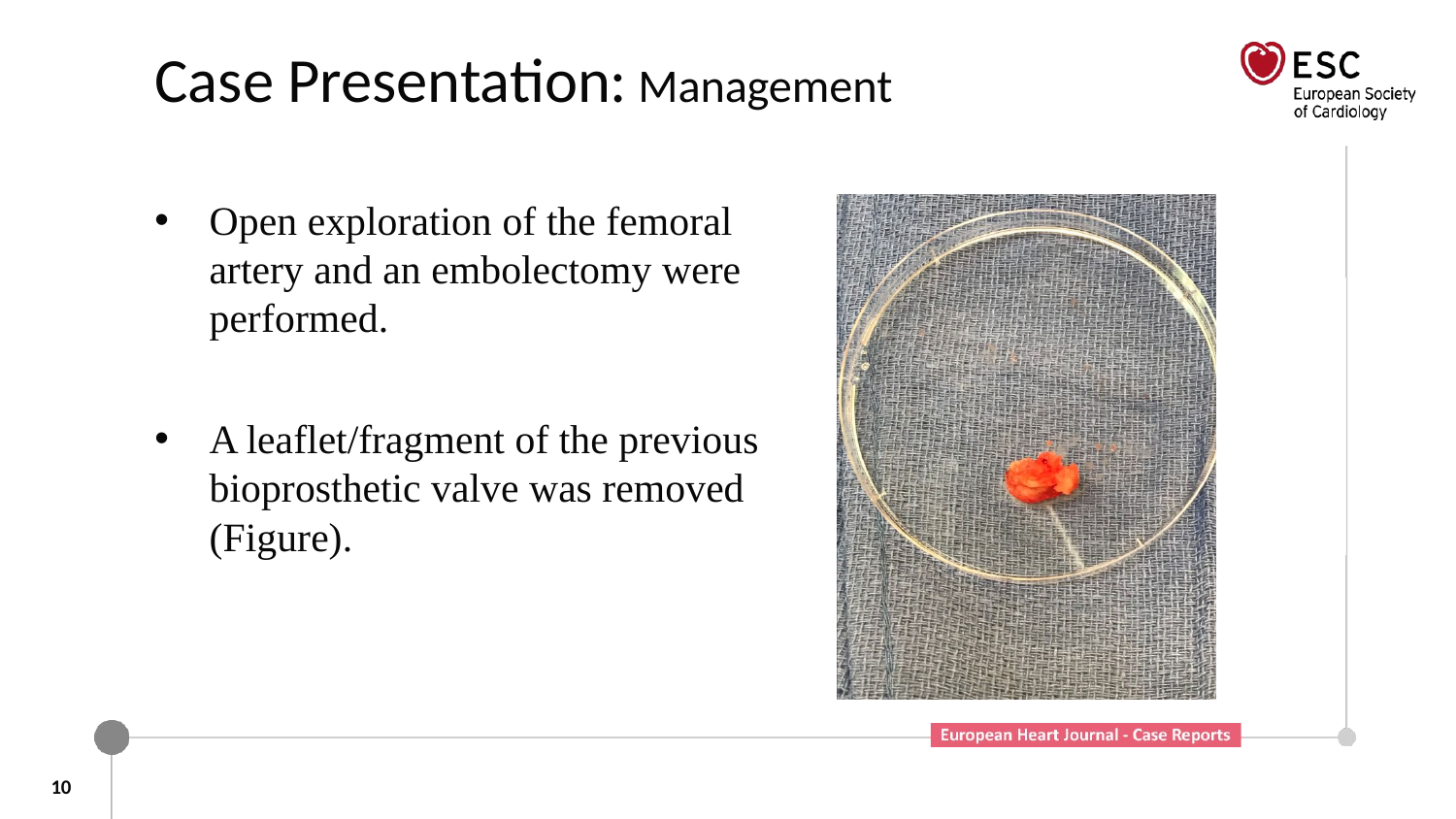

# Case Presentation: Management
Open exploration of the femoral artery and an embolectomy were performed.
A leaflet/fragment of the previous bioprosthetic valve was removed (Figure).
10

## Slide 11
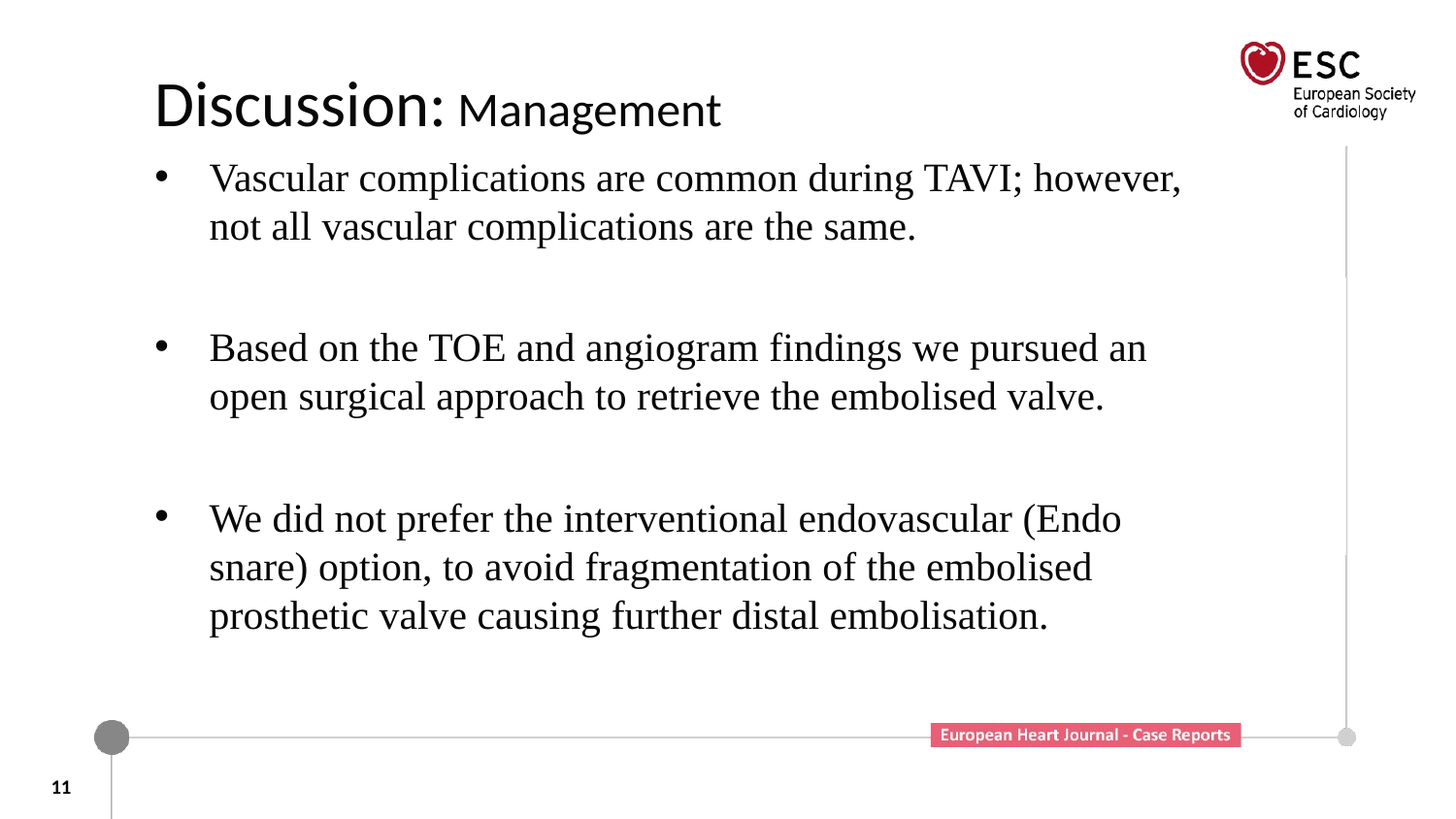

# Discussion: Management
Vascular complications are common during TAVI; however, not all vascular complications are the same.
Based on the TOE and angiogram findings we pursued an open surgical approach to retrieve the embolised valve.
We did not prefer the interventional endovascular (Endo snare) option, to avoid fragmentation of the embolised prosthetic valve causing further distal embolisation.
11

## Slide 12
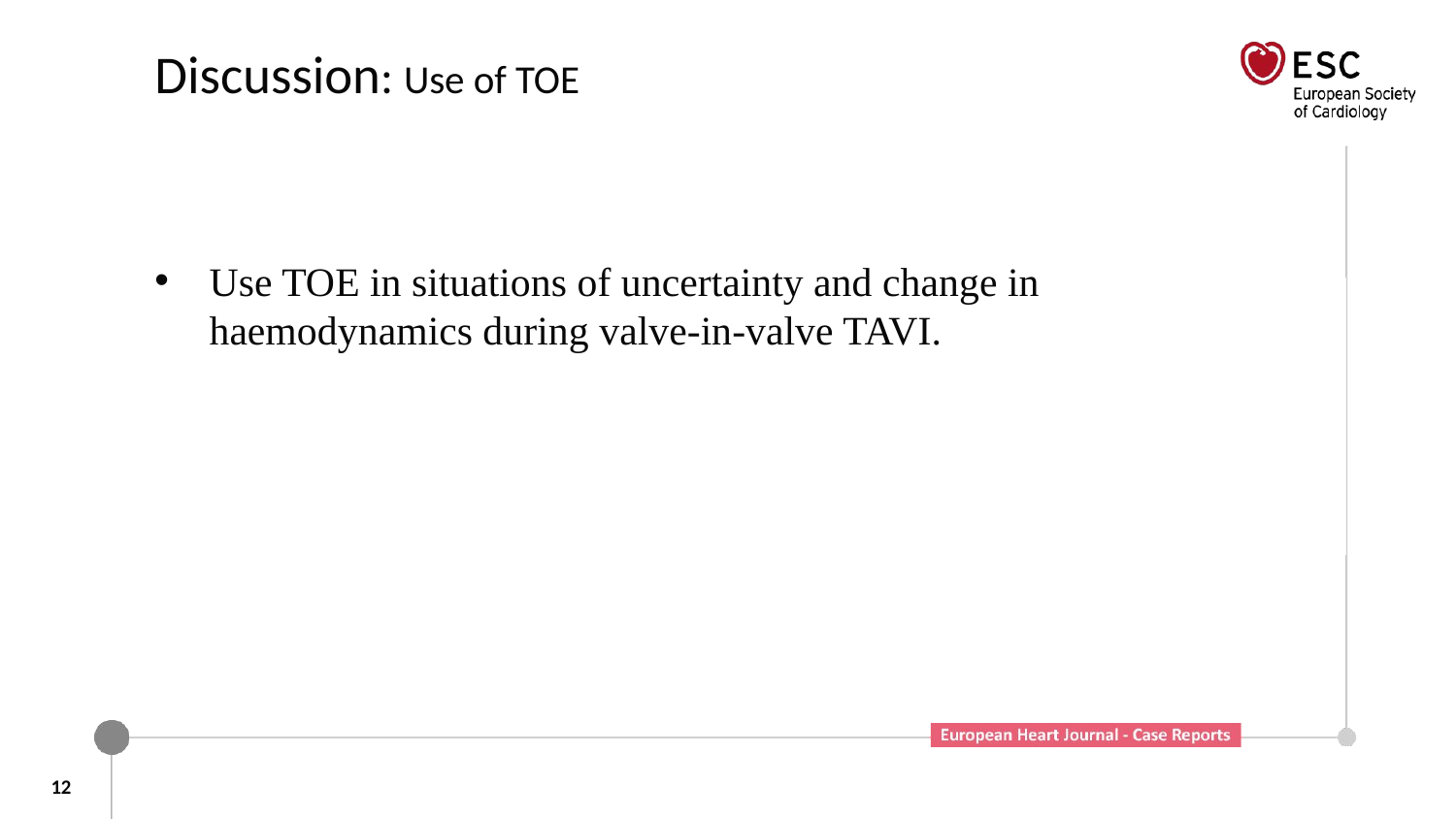

# Discussion: Use of TOE
Use TOE in situations of uncertainty and change in haemodynamics during valve-in-valve TAVI.
12

## Slide 13
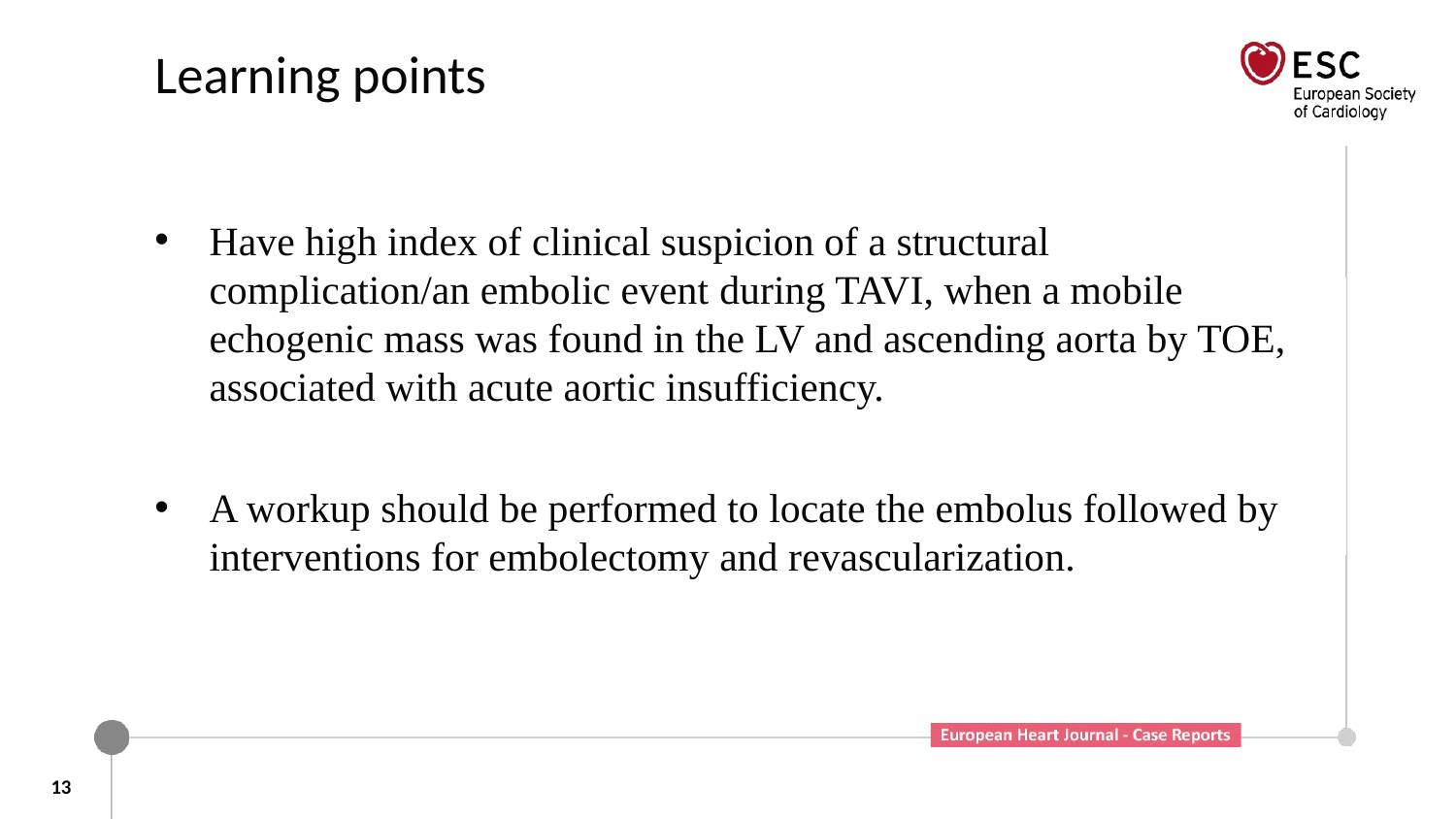

# Learning points
Have high index of clinical suspicion of a structural complication/an embolic event during TAVI, when a mobile echogenic mass was found in the LV and ascending aorta by TOE, associated with acute aortic insufficiency.
A workup should be performed to locate the embolus followed by interventions for embolectomy and revascularization.
13

## Slide 14
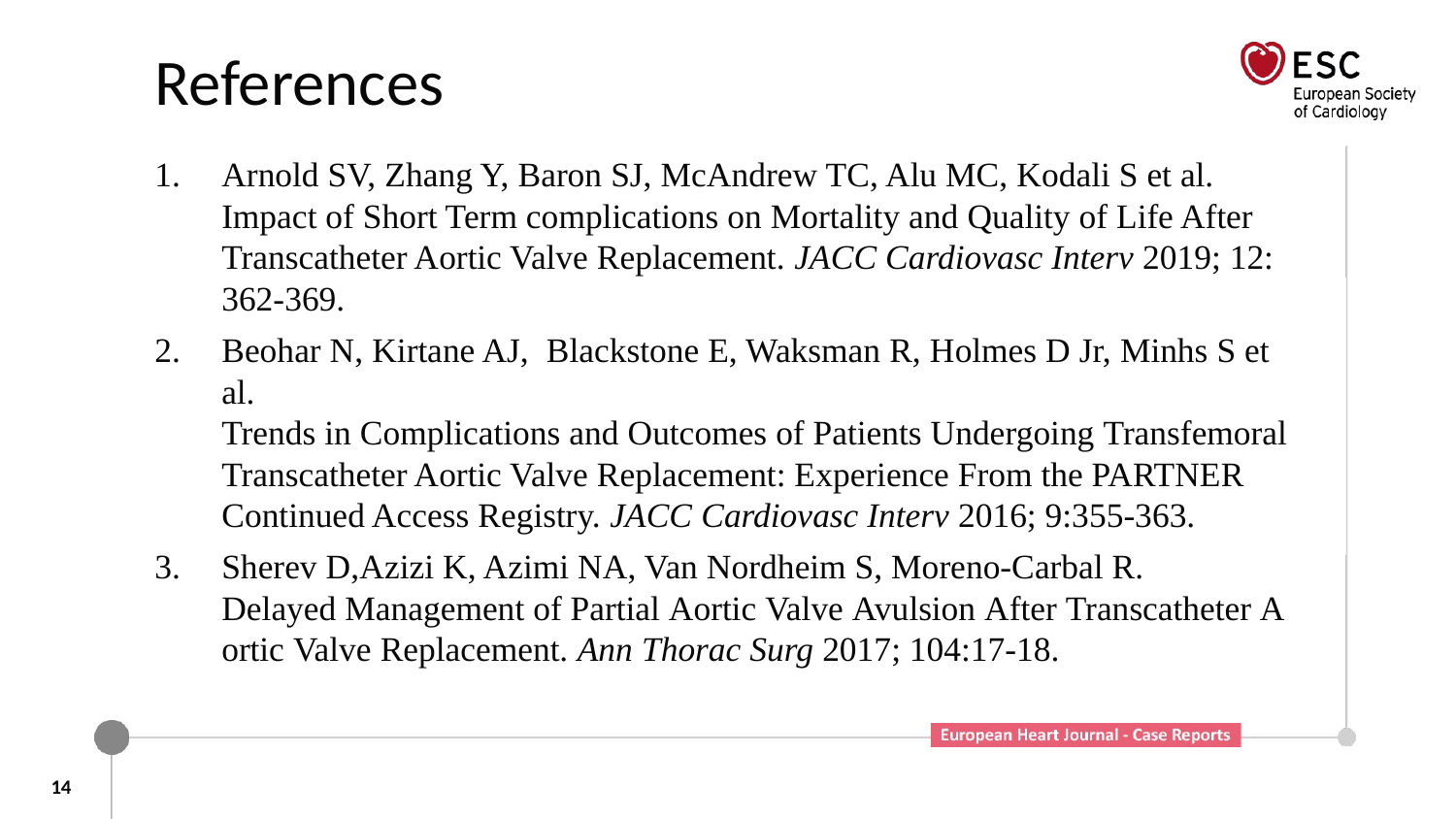

# References
Arnold SV, Zhang Y, Baron SJ, McAndrew TC, Alu MC, Kodali S et al. Impact of Short Term complications on Mortality and Quality of Life After Transcatheter Aortic Valve Replacement. JACC Cardiovasc Interv 2019; 12: 362-369.
Beohar N, Kirtane AJ, Blackstone E, Waksman R, Holmes D Jr, Minhs S et al. Trends in Complications and Outcomes of Patients Undergoing TransfemoralTranscatheter Aortic Valve Replacement: Experience From the PARTNER Continued Access Registry. JACC Cardiovasc Interv 2016; 9:355-363.
Sherev D,Azizi K, Azimi NA, Van Nordheim S, Moreno-Carbal R. Delayed Management of Partial Aortic Valve Avulsion After Transcatheter Aortic Valve Replacement. Ann Thorac Surg 2017; 104:17-18.
14
